# Tretinoin improves the anti-cancer response to cyclophosphamide, in a model-selective manner

**DOI:** 10.1186/s12885-024-11915-5

**Published:** 2024-02-13

**Authors:** Caitlin M. Tilsed, M. Lizeth Orozco Morales, Rachael M. Zemek, Brianna A. Gordon, Matthew J. Piggott, Anna K. Nowak, Scott A. Fisher, Richard A. Lake, W. Joost Lesterhuis

**Affiliations:** 1 National Centre for Asbestos Related Diseases, 6009 Nedlands, WA Australia; 2 School of Biomedical Sciences, University of Western Australia, 6009 Crawley, WA Australia; 3 Institute for Respiratory Health, 6101 Perth, WA Australia; 4 Telethon Kids Institute, University of Western Australia, 6872 West Perth, WA Australia; 5 School of Molecular Sciences, University of Western Australia, 6009 Crawley, WA Australia; 6 Department of Medical Oncology, Sir Charles Gairdner Hospital, 6009 Nedlands, WA Australia

**Keywords:** Cyclophosphamide, Tretinoin, Interferon, Sensitisation, Combination treatment

## Abstract

**Background:**

Chemotherapy is included in treatment regimens for many solid cancers, but when administered as a single agent it is rarely curative. The addition of immune checkpoint therapy to standard chemotherapy regimens has improved response rates and increased survival in some cancers. However, most patients do not respond to treatment and immune checkpoint therapy can cause severe side effects. Therefore, there is a need for alternative immunomodulatory drugs that enhance chemotherapy.

**Methods:**

We used gene expression data from cyclophosphamide (CY) responders and non-responders to identify existing clinically approved drugs that could phenocopy a chemosensitive tumor microenvironment (TME), and tested combination treatments in multiple murine cancer models.

**Results:**

The vitamin A derivative tretinoin was the top predicted upstream regulator of response to CY. Tretinoin pre-treatment induced an inflammatory, interferon-associated TME, with increased infiltration of CD8 + T cells, sensitizing the tumor to subsequent chemotherapy. However, while combination treatment significantly improved survival and cure rate in a CD4^+^ and CD8^+^ T cell dependent manner in AB1-HA murine mesothelioma, this effect was model-selective, and could not be replicated using other cell lines.

**Conclusions:**

Despite the promising data in one model, the inability to validate the efficacy of combination treatment in multiple cancer models deprioritizes tretinoin/cyclophosphamide combination therapy for clinical translation.

**Supplementary Information:**

The online version contains supplementary material available at 10.1186/s12885-024-11915-5.

## Background

Although chemotherapy is used to treat many advanced or metastatic cancers, it is rarely curative, and is predominantly palliative. With the emergence and success of immune checkpoint therapy (ICT) in a wide range of cancers as a combination or monotherapy [1–3], there has been a shift towards investigating combinations of chemotherapy with immunotherapy to further improve the efficacy over each treatment alone. Combination chemoimmunotherapy has shown promise in mesothelioma^4^ and lung cancer [4, 5], with the addition of anti-PD1 antibodies increasing the response rate to cisplatin and pemetrexed, or platinum and etoposide chemotherapy, respectively. While these regimes are more effective than monotherapy, a large proportion of patients still do not respond. Additionally, ICT, particularly dual ICT with anti-PD1 and anti-CTLA-4, can lead to severe side effects [6] including hyperprogressive disease [7]. Therefore, alternative immunomodulatory drugs need to be identified and their additive or synergistic effects with chemotherapy established.

We previously characterised a gene expression signature associated with complete response to cyclophosphamide (CY) chemotherapy [8]. We, and others, found that an inflammatory, CD4^+^ T cell-associated tumor microenvironment (TME) is necessary for response to chemotherapy in both mice and patients [8–10]. A potential avenue for increasing the effectiveness of chemotherapy would be to identify key drivers of this chemo-sensitive phenotype and therapeutically target them to upregulate expression and remodel the TME, using repurposed drugs. This approach was utilised in the context of ICT [11, 12]. Network analysis of gene expression data from responding and non-responding mice identified tretinoin as a key promoter of an ICT sensitive tumor. In vivo validation in mesothelioma and sarcoma murine models showed a significant increase in anti-CTLA-4 efficacy when tretinoin was co-administered, demonstrating that this experimental strategy can identify drugs that can be repurposed for cancer treatment and synergise with existing therapies [11, 12].

Tretinoin, also known as all-*trans*-retinoic acid, is a pan-retinoic acid receptor (RAR) agonist and an active metabolite of vitamin A. Retinoids signal via nuclear receptors to regulate cell growth, differentiation and apoptosis [13, 14]. Tretinoin has been shown to have potent anti-cancer activity in vitro [15, 16] and in vivo [17, 18], and is the first-line treatment for acute promyelocytic leukemia, inducing complete remission in > 90% of patients, when given as monotherapy or in combination with chemotherapy [19, 20]. A randomized phase II trial showed that coadministration of tretinoin with cisplatin and paclitaxel in patients with non-small cell lung cancer increased both response rate and overall survival [21], and was well tolerated when combined with gemcitabine and paclitaxel in pancreatic cancer [22], and with paclitaxel in breast cancer [23].

Aside from its direct anti-tumor capability, tretinoin has a wide range of immunomodulatory effects [24–26]. In particular, it is a powerful regulator of T cell activity and is capable of: inducing Foxp3 expression in peripheral CD4^+^ T cells, and polarising them towards a more suppressive T regulatory cell (Treg) phenotype [27, 28]; increasing Treg infiltration into the tumor [18]; polarizing CD4^+^ T cells towards different T helper subsets; and promoting CD4 effector responses [29, 30]. Furthermore, tretinoin enhances the survival, expansion and activation of CD8^+^ T cells [25, 26, 31, 32].

While the role of tretinoin in mediating immunity has been well studied, there are limited preclinical studies investigating whether it can be repurposed as an immunotherapy and combined with classical chemotherapy to improve treatment efficacy. Here, we used gene expression data from responding and non-responding tumors, harvested from mice treated with CY, to identify drugs that could be repurposed for administered alongside chemotherapy to improve response rates. Tretinoin was the top candidate identified from upstream regulator analysis and synergised with CY in AB1HA murine mesothelioma, significantly improving survival, however, in a model-selective manner.

## Methods

### Mice

Male or female 8–10 week-old BALB/cArc or C57BL/6 mice were obtained from the Animal Resources Centre (Murdoch, Western Australia) and housed in pathogen free conditions. All experiments were conducted with animal ethics approval from the Harry Perkins Institute Animal Ethics Committee (AE099 and AE153).

### Cell lines and inoculation

AB1-HA, AE17, CT26, WEHI164 and 4T1 were obtained from Cell Bank Australia (CBA) and maintained in RPMI 1640 supplemented with 20 mM HEPES, 0.05 mM 2-mercaptoethanol, 100 U/ml benzylpenicillin, 50 µg/ml gentamycin and 10% NCS. AB1HA was additionally cultured with G418 to maintain expression of HA. LLC was obtained from CBA and maintained in DMEM (Gibco) supplemented as described above.

Cells were grown to a confluence of 80% and passaged a minimum of three times prior to inoculation. Cells were collected using trypsinisation and a viability of at least 90% was confirmed using trypan blue exclusion. 5 × 10^5^ cells were inoculated subcutaneously in 100 µl PBS. Tumour growth was monitored using callipers and size in mm^2^ was calculated from perpendicular length and width measurements using the formula; Tumour area (mm^2^) = length (mm) x width (mm).

### In vivo treatments

Cyclophosphamide (Endoxan) was obtained from the Sir Charles Gardiner Hospital pharmacy (Perth, Australia) and dosed at 200 mg/kg. Chemotherapy drugs were prepared under sterile conditions and diluted in 0.9% saline solution. Drugs were administered intraperitoneally (i.p) at a maximum volume of 100 µl per 10 g weight of mouse.

All-*trans*-retinoic acid (≥ 98% by HPLC, powder) was purchased from Sigma-Aldrich (Macquarie Park, NSW) and suspended in dimethyl sulfoxide (DMSO; Sigma-Aldrich) at a concentration of 40 mg/ml for short term storage at − 80˚C. Mice were treated with 10 mg/kg of tretinoin for 9 consecutive days, intraperitoneally. Working concentrations of tretinoin were suspended in PBS and had a final DMSO concentration of 5%.

For all immune cell depletion experiments, antibody administration began three days before tumour inoculation. Anti-CD4 (clone GK1.5) and anti-CD8 (clone YTS 169; both BioXcell, New Hampshire, USA) were dosed i.p at 100 µg in 100 µl of PBS on day 3, day 0 and thereafter weekly until the end of experiment. Depletion was monitored in peripheral blood collected from the tail vein and analysed by flow cytometry.

### RNAseq

The transcriptomic profile of responders and non-responders was generated in a previous study [8]. Briefly, a bilateral tumour model was to surgically resect one tumour for analysis, with the remaining tumour providing a read-out for response or non-response to CY. RNA was extracted using RNeasy MinElute columns (QIAGEN) and sequencing (Novaseq6000 20 million reads, 100 bp single end) was performed by the Australian Genome Research Facility. Data were aligned using Kalisto, [33] and differential expression analysis was performed using DESeq2 [34]. For analysis comparing responders and non-responders to CY, a FDR of < 0.1 (BenjaminiHochberg method, B-H) was considered significant. Upstream regulator analysis was performed using Ingenuity [35] and the top 10 clinically approved drugs by significance were selected. RNA sequencing was similarly performed on untreated AE17 [36], CT26 [37] and WEHI164 [37].

Gene set enrichment analysis (GSEA) [38] was performed using curated gene sets. The first, ‘tretinoin induced’ gene set was created by selecting the DEG upregulated by tretinoin treatment using data generated in a previous study [12]. The second was obtained from Kang et al. [39]; CD4^+^ T cells were cultured in the presence or absence of RA and genes upregulated in response to treatment collated into the ‘tretinoin treated CD4^+^ T cells Kang 2011’ gene set. The enrichment of these gene sets was assessed in complete responders or progressors to CY from Tilsed et al. [8]. A positive enrichment indicated the gene set was enriched in complete responders and a negative enrichment score indicated the genes were enriched in progressors. The GSEA ‘inflammatory response’ hallmark gene set was used to generate a heatmap displaying the relative expression level of inflammatory genes within each cell line. The inflammatory score was calculated by averaging the normalized counts [40] of the genes in the GSEA hallmark ‘inflammatory response’ geneset. Immune scores were calculated using ESTIMATE [41], which performs single-sample GSEA to infer the level of infiltrating immune cells.

### MTT assays

MTT assays were performed to assess cell viability via metabolic activity. In short, cells were seeded at 1 × 10^3^ cells/well in a 96 well plate. The cells were incubated overnight before serial dilutions of each drug were added in triplicate and incubated for 48 h at 5% CO_2_ at 37 °C. Acrolein was freshly distilled and stored at -80 °C until use; purity was confirmed by ^1^H NMR spectroscopy. Mafosfamide cyclohexylamine (mafosfamide) was purchased from Niomech (Bielefeld, Germany). Cells were then treated with 50 µl of 2 mg/ml 3-[4,5-dimethylthiazol-2-yl]-2,5-diphenyltrtretinoinzolium bromide (MTT). Four hours later, all the medium was aspirated from the wells and the plate was centrifuged. The formazan crystals were dissolved in 100 µl DMSO and absorbance was measured at 570 nm using a SpectraMax M5e (Molecular Devices).Cell viability was determined using the blank corrected absorbance values and untreated cells as a negative control: % viability = ($\frac{\text{a}\text{b}\text{s}\text{o}\text{r}\text{b}\text{a}\text{n}\text{c}\text{e} \text{o}\text{f} \text{e}\text{x}\text{p}\text{e}\text{r}\text{i}\text{m}\text{e}\text{n}\text{t}\text{a}\text{l} \text{w}\text{e}\text{l}\text{l}}{\text{a}\text{b}\text{s}\text{o}\text{r}\text{b}\text{a}\text{n}\text{c}\text{e} \text{o}\text{f} \text{u}\text{n}\text{t}\text{r}\text{e}\text{a}\text{t}\text{e}\text{d} \text{c}\text{o}\text{n}\text{t}\text{r}\text{o}\text{l}})\times 100$.

## Results

### Tretinoin increases the efficacy of cyclophosphamide in AB1-HA murine mesothelioma

**Fig. 1:**
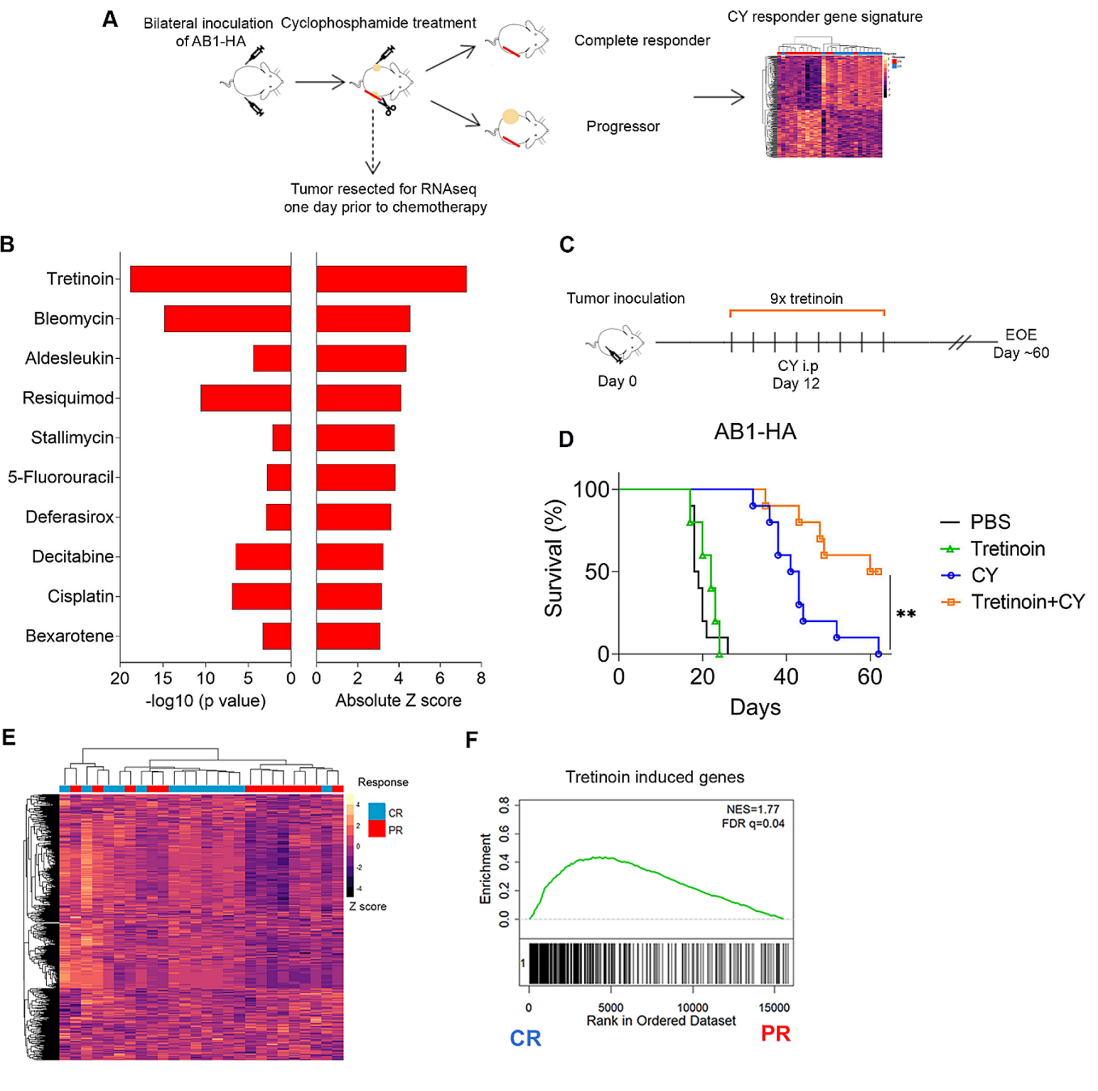
Tretinoin sensitises AB1-HA murine mesothelioma to cyclophosphamide chemotherapy. **(A)**. The generation of the cyclophosphamide (CY) responder signature. Tumors from complete responders and progressors were sequenced and the differentially expressed genes upregulated in complete responders were made into a ‘CY responder gene signature’ (*n* = 178 genes). **(B)** Drugs predicted to induce a CY-sensitive tumor microenvironment, as determined by Ingenuity upstream regulatory analysis. **(C)** Experimental design for tretinoin and CY combination treatment. Mice were inoculated with AB1-HA s.c. and treated with tretinoin i.p. starting 3 days prior to cyclophosphamide and continued for 9 total doses at 10 mg/kg. CY was dosed at 200 mg/kg on day 12. **(D)** Survival of mice inoculated with AB1-HA and treated with CY, tretinoin or the combination (*n* = 10) (**E**) Heatmap of complete responders (CR) and progressors (PR) to CY treatment clustered using a ‘tretinoin induced genes’ set. (**F**) Gene set enrichment analysis of tretinoin associated gene sets in complete CY responders or progressors. Positive NES indicated a gene set is enriched in complete responders. Significance was determined using the Mantel-Cox log-rank test. *p < 0.05 **p < 0.01

In a previous study, we found that a pre-treatment inflamed and immune infiltrated ‘hot’ tumor microenvironment (TME) was associated with a complete response to cyclophosphamide (CY) chemotherapy in both mice and patients [8]. From these data, we generated an IFN, TNFα CD4^+^ T cell gene signature that predicts response to CY (Fig. 1A). We performed upstream regulator analysis to identify drugs that could induce the gene expression signature associated with chemotherapy responsive tumours (Fig. 1B). We focused on drugs that are clinically approved that could be repurposed and combined with CY in a cancer setting. The top upstream regulator predicted to induce a CY sensitive TME was tretinoin. Another activator of the retinoic acid signalling pathway, bexarotene was also in the top ten candidate drugs as determined by z score and *p* value.

As tretinoin was the highest ranked upstream regulator, and has both direct anti-tumor [15, 16] and immunomodulatory [24] effects, we investigated whether the addition of tretinoin to CY increased response rates or survival in AB1-HA murine mesothelioma. Tumour bearing mice were treated daily with systemic tretinoin, commencing 3 days before chemotherapy for a total of 9 doses (Fig. 1C). The addition of tretinoin significantly increased survival after cyclophosphamide treatment (Fig. 1D, *p* = 0.0052) and improved the complete response rate from 0 to 50%. The addition of tretinoin to CY was well tolerated, with no additional weight loss observed compared to CY alone (Supplementary Fig. 1). These data demonstrate that upstream regulators of a chemotherapy-sensitive TME can be identified from transcriptomic data and validated in vivo, as the addition of tretinoin to CY was significantly beneficial, curing tumours in a context where each treatment was unable to cure as monotherapy (Fig. 1D).

### Genes upregulated by tretinoin are associated with responders to CY

As we observed that the addition of tretinoin to CY improved the response rate, we were interested in whether this was due to the induction of a CY sensitive TME. In a previous study, we characterised the transcriptomic changes to the TME induced by tretinoin [12]. In this study, mice were inoculated with AB1-HA and treated with systemic tretinoin for five days upon which the tumors were collected and sequenced. We then performed differential gene expression analysis to identify the genes upregulated by tretinoin to construct a ‘tretinoin induced’ gene set. We took these ‘tretinoin induced’ genes and looked at their enrichment in complete responders and progressors to CY. We were able to distinguish between complete responders and progressors to CY using unsupervised hierarchical clustering (Fig. 1E). Using gene set enrichment ^39^ we also observed enrichment of these genes in responders compared to progressors (Fig. 1F), indicating that the genes upregulated by tretinoin treatment are similar to those required for a response to CY.

### Tretinoin does not increase the sensitivity of tumor cells to cyclophosphamide in vitro

**Fig. 2:**
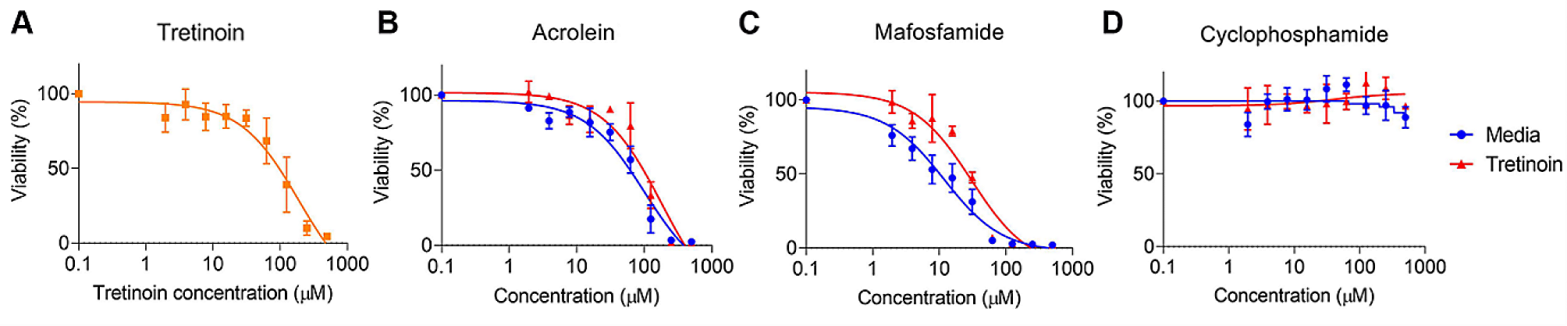
Tretinoin does not increase the sensitivity of AB1-HA to cyclophosphamide or its metabolites in vitro. (**A**) Viability of AB1-HA cells treated with increasing concentrations of tretinoin. **(B-D)**. Viability of AB1-HA treated with increasing concentrations of acrolein **(B)**, mafosfamide **(C)** or cyclophosphamide **(D)** with or without 50 µM of tretinoin. Viability was determined by the MTT assay. Data points are the mean ± SD of three independent experiments

Along with having immune-mediated and direct cytotoxic effects, tretinoin can also enhance the chemosensitivity of tumor cells in vitro [42–44]. To determine whether tretinoin enhanced chemosensitivity in AB1-HA, we performed MTT assays using CY and its metabolite acrolein or the cyclofosfamide analogue mafosfamide, which spontaneously degrades to 4-hydroxycyclophosphamide [45], in combination with tretinoin. A noncytotoxic dose of 50 µM tretinoin (Fig. 2A) was added in combination with each drug. The addition of tretinoin to the CY or its metabolite acrolein (*p* = 0.56) or mafosfamide (*p* = 0.27) did not significantly alter the IC_50_ values for these compounds (Fig. 2BD), demonstrating that tretinoin does not increase the inherent sensitivity of tumor cells to the drugs. This suggests that tretinoin has a non-cancer cell-mediated mechanism of action that drives its enhancement of CY efficacy in AB1-HA.

### The effectiveness of combination tretinoin–CY is dependent on both CD4^+^ and CD8^+^ T cells

**Fig. 3:**
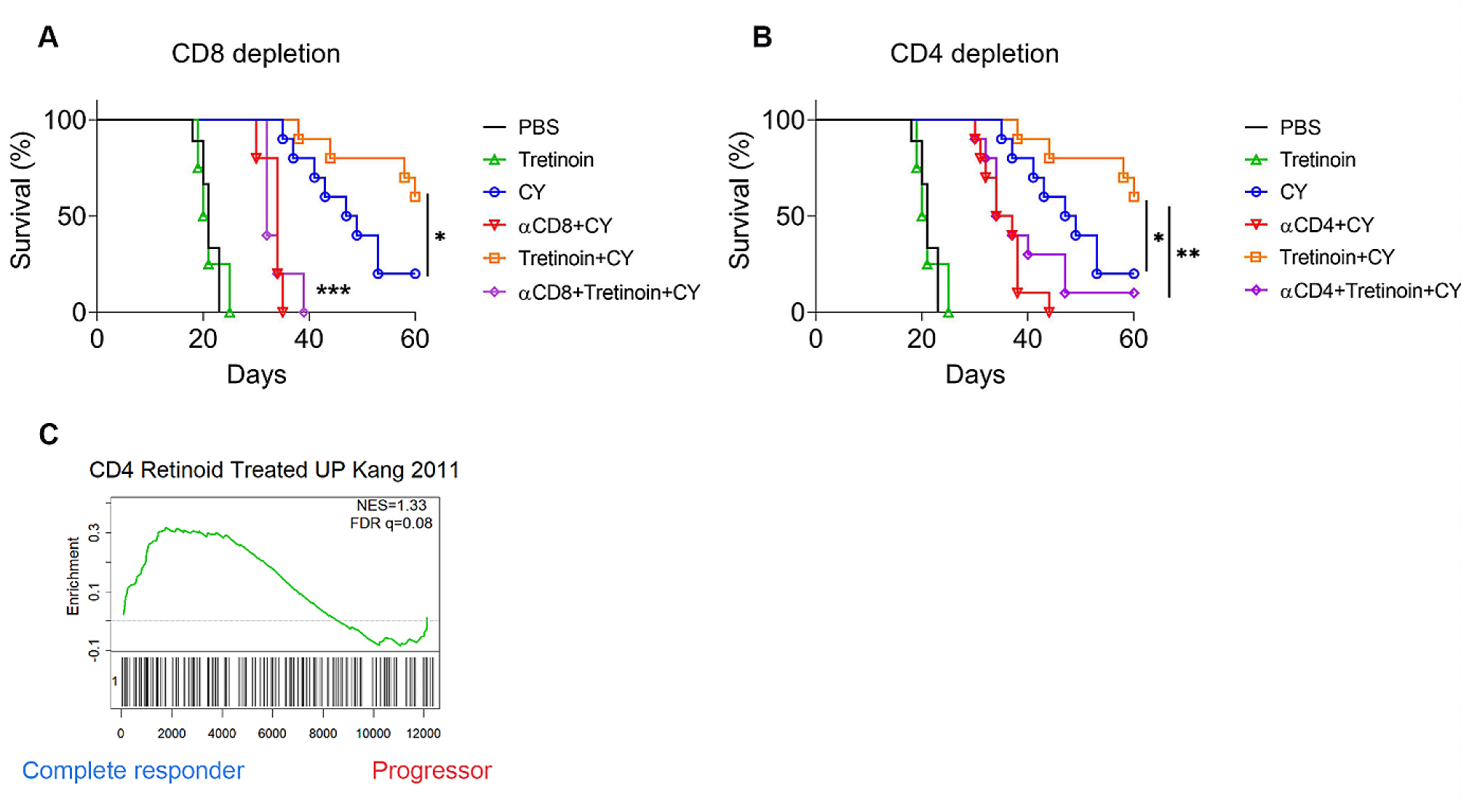
The efficacy of combination tretinoin–CY treatment is both CD8^+^ and CD4^+^ T cell dependent. **(A-B)** Survival of mice inoculated with AB1-HA and treated with 200 mg/kg CY with or without tretinoin. Mice were depleted of CD8 (B) or CD4 (C) T cells using 100 µg αCD4 or αCD8 i.p., 3days prior to inoculation and depletion was maintained throughout the experiment. Mice were dosed daily with 10 mg/kg tretinoin, beginning 3 days before CY for nine total doses. (**C**) Enrichment analysis of CD4 tretinoin-associated gene set in complete responders or progressors to CY. Positive NES indicates a gene set is enriched in CR. Significance for survival analysis was determined using the Mantel-Cox log-rank test. *p < 0.05 **p < 0.01. *n* = 5 for tretinoin, αCD8 + CY and αCD8 + CY + tretinoin, *n* = 10 for all other groups

As tretinoin has been reported to have a variety of effects on the immune system and cyclophosphamide efficacy is dependent on both CD8^+^ and CD4^+^ T cells [8, 46, 47], we next asked whether the combination of tretinoin and CY was also T cell dependent, or whether the addition of tretinoin could overcome the absence of either immune component. Mice were depleted of CD4^+^ or CD8^+^ T cells prior to inoculation with AB1HA, with depletion maintained for the duration of the experiment. Tretinoin and CY were administered as in Fig. 1. The depletion of both CD4^+^ (*p* = 0.003) or CD8^+^ T cells (*p* = < 0.0001) significantly decreased the efficacy of the tretinoin–CY combination treatment, with 1/10 or 0/5 mice cured, respectively, compared to 6/10 in immunocompetent mice (Fig. 3A-B).

To further explore the link between tretinoin and T cells we used a gene set derived from CD4^+^ T cells treated with tretinoin [39] to determine whether tretinoin induced changes to CD4^+^ T cells are also associated with CY response. The genes upregulated by tretinoin in CD4^+^ T cells were also enriched in complete responders to CY (Fig. 3C), indicating that tretinoin may induce phenotypic changes to CD4^+^ T cells that lead to improved response to CY. These data demonstrate that the efficacy of the tretinoin and CY combination is T cell dependent.

### Tretinoin sensitization to cyclophosphamide efficacy is dependent on the cancer model used

**Fig. 4:**
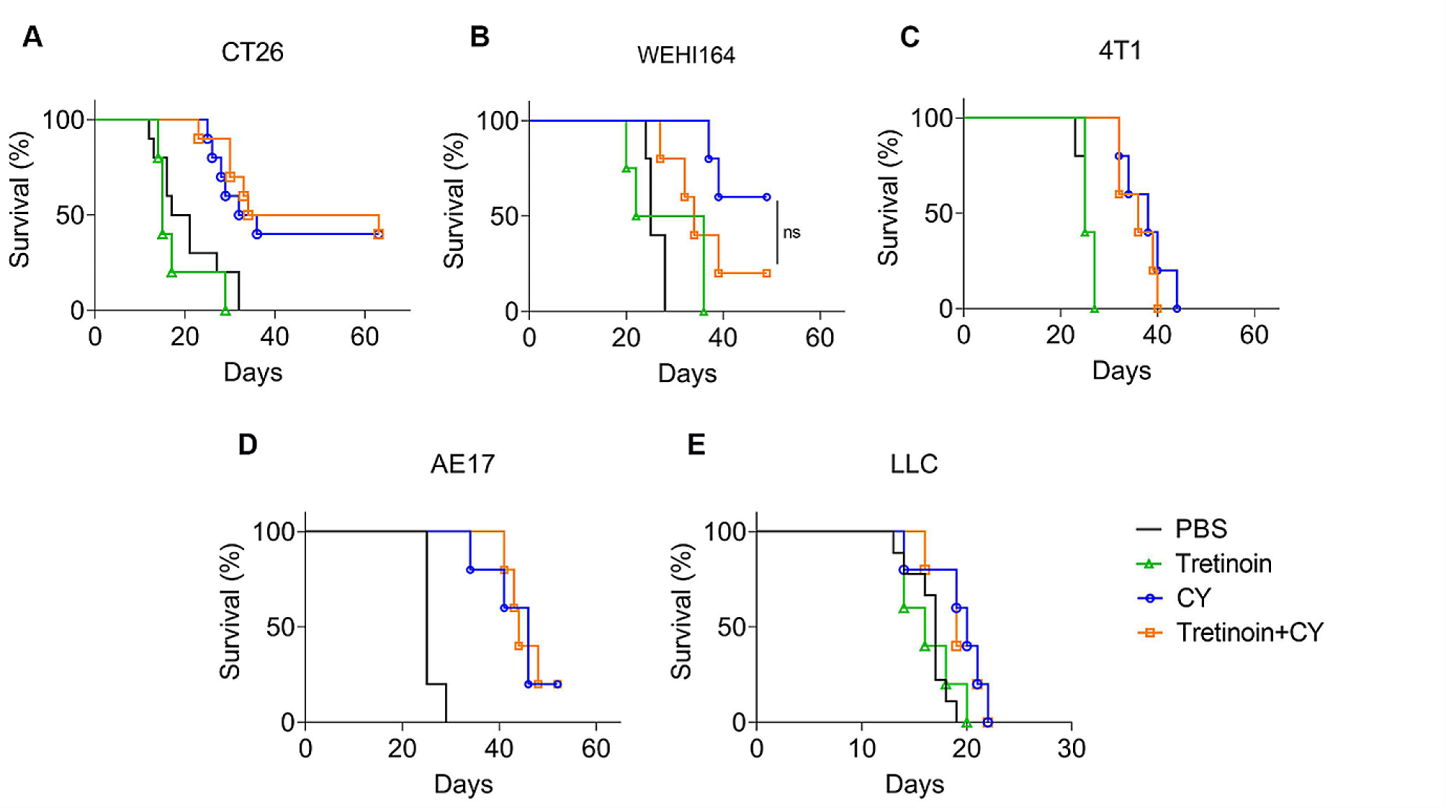
Tretinoin does not increase the efficacy of cyclophosphamide in alternative cell lines **(A–E)**. Tumor bearing mice were treated with tretinoin 3 days before CY, for a total of nine doses at 10 mg/kg. CY was dosed at 200 mg/kg. Survival of mice inoculated with **(A)** CT26 colon carcinoma (*n* = 10, *n* = 5 for tretinoin group), **(B)** WEHI164 sarcoma (*n* = 5), **(C)** 4T1 breast cancer (*n* = 5), **(D)** AE17 murine mesothelioma (*n* = 5) or **€** Lewis lung carcinoma (*n* = 5, *n* = 10 for PBS). Significance was determined using the Mantel-Cox log-rank test. “ns” = not significant

As we observed that the addition of tretinoin significantly increased CY efficacy in AB1HA mesothelioma, we tested whether this treatment worked in other cancer models. The same treatment schedule was used with mice inoculated with CT26 colon carcinoma, WEHI164 sarcoma, 4T1 breast cancer, AE17 murine mesothelioma, or Lewis Lung Carcinoma (LLC) cell lines. Despite cyclophosphamide as a monotherapy delaying tumor growth and improving survival in all cell lines (Supplementary Table 1), the addition of tretinoin had no effect on survival or tumor growth (Fig. 4), indicating only the combination of tretinoin and CY is model selective.

### In vivo cancer models have varying expression of RARs and RXRs which may drive differing sensitivity to tretinoin

**Fig. 5:**
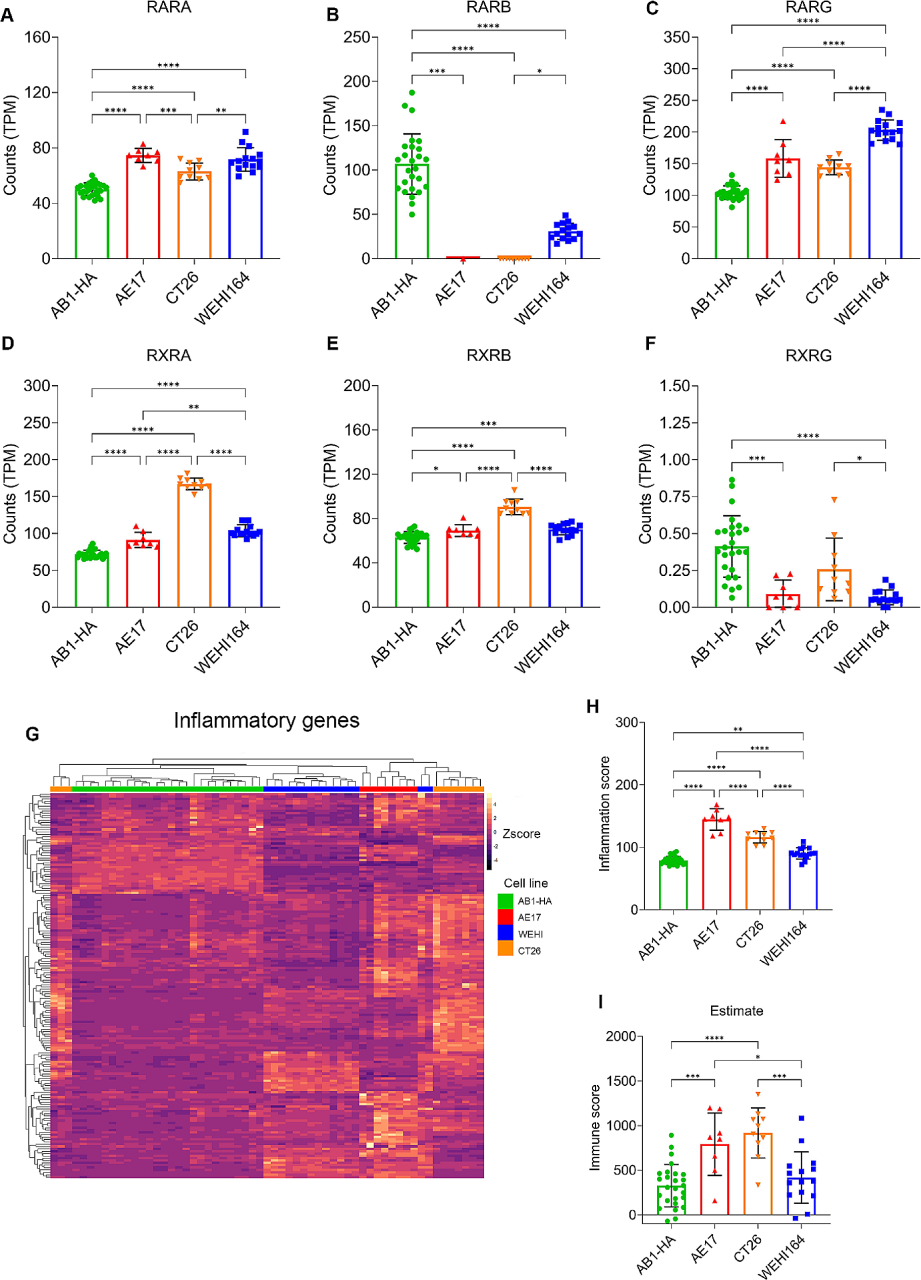
The expression of retinoic acid receptors and inflammatory genes in AB1-HA, AE17,CT26 and WEHI164 from bulk RNA sequencing data. Tumors from mice inoculated with AB1HA, AE17,CT26 or WEHI164 were harvested, and RNA was extracted and sequenced. **(A–C)** Expression of the three retinoic acid receptors. **(D–F)** Expression of the three retinoic X receptors. Counts were normalised to adjust for sequencing depth. (**G**) Heatmap of inflammatory genes. Counts were normalized and z scaled. (**H**) Inflammatory score of each tumor type, as calculated by taking the average expression of inflammation associated genes in each sample. (**H**) Immune score for each tumor as determined by ESTIMATE. Data are shown as mean ± SD and significance was determined using Mann-Whitney U tests corrected for multiple comparisons *p < 0.05, **p < 0.01 ***p < 0.001. *n* = 26 for AB1-HA, *n* = 8 for AE17, *n* = 10 for CT26 and *n* = 15 for WEHI164.

As we saw such a robust anti-tumor response to the combination of tretinoin and CY in the AB1-HA model but not in models using other cell lines, we tested whether the expression of retinoic acid receptors (RARs) or retinoid X receptors (RXRs) varied between tumor models, as a potential explanation of the differing outcomes. Retinoic acid signals through RARs or RXRs and it has been previously reported that the expression or induction of these receptors can influence sensitivity to tretinoin [16]. To examine this in our cancer models, we compared the expression of RARα, RARβ, RARγ, RXRα, RXRβ and RXRγ in AB1-HA tumors, which are sensitive to combination tretinoin–CY therapy, and AE17, CT26 and WEHI164 tumors, which are resistant. AB1-HA tumors expressed lower levels of RARα, RARγ, RXRα and RXRβ compared to all other cell lines (Fig. 5B). Only RARβ and RXRγ were expressed at higher levels in AB1-HA tumors (Fig. 5). The differing expression of RARS and RXRS may contribute to the difference in sensitivity between AB1-HA, which is sensitive to our treatment regime, and AE17, CT26 WEHI164 which are comparably treatment resistant.

### The expression of inflammatory genes in the TME is not associated with response

Lastly, we investigated whether the difference in the sensitivity of our tumor models to tretinoin and CY could be attributed to the level of inflammation and immune infiltration which are known characteristics of a chemotherapy-sensitive TME [8, 48]. Using bulk RNAseq data from AB1-HA, AE17, CT26 and WEHI164 [8, 36, 37], we plotted the GSEA hallmark ‘inflammatory response’ gene set across models (Fig. 5G). AB1-HA and WEHI had a lower expression of inflammatory genes compared to AE17 and CT26, as well as a lower inflammatory score (Fig. 5H) and immune score (Fig. 5I). These results demonstrate that there is no association between the level of inflammation in the TME and the response to tretinoin and CY.

## Discussion

As combination or sequential chemotherapies are frequently not curative in advanced cancers, there has been a movement towards combining chemotherapy with other treatments, such as immunotherapy, to increase response rates and improve patient outcomes [4, 5, 49]. We identified tretinoin as the top predicted regulator of a chemosensitive TME using data previously generated from sequencing the tumors of responders and non-responders to CY [8]. Tretinoin is directly cytotoxic and can normalise cancerous cells [15, 50], but also has potent immunomodulatory effects [12, 24], making it a prime candidate to combine with chemotherapy. We tested the ability of tretinoin to improve the response to CY as a pre-treatment, hypothesising that tretinoin would induce a TME that is sensitive to subsequent CY treatment. Indeed, we found the addition of tretinoin to be strongly synergistic, increasing CY efficacy in AB1HA murine mesothelioma. However, efficiency did not extend to other cancer models, indicating that there may be intrinsic characteristics of the AB1-HA tumor model that drive sensitivity to this treatment regime.

The high variability in responsiveness to immunotherapy between mouse cancer models has been well-documented but remains poorly understood [51–53]. Indeed, others have also found mixed efficacy from the combination of tretinoin and CY in preclinical models. In a murine model of metastatic rhabdomyosarcoma, the addition of tretinoin to CY decreased the number of metastatic liver nodules 4.8 fold and improved survival [54]. However, in mice inoculated with TC1 myeloma, the combination had no additional benefit over the monotherapies [55]. Tretinoin also demonstrated synergy when combined with gemcitabine in murine pancreatic adenocarcinoma, decreasing hypoxia, invasion and overall tumor burden [56]. However, the clinical success of this combination has been limited and any synergistic effects limited to acute promyelocytic leukaemia, and phase II observations in non-small cell lung cancer [21]. While the coadministration of tretinoin and paclitaxel and/or paclitaxel has been well tolerated in breast cancer [23] and pancreatic cancer [22, 57], there was no additional benefit over the chemotherapy-only control arm.

In vitro, tretinoin has been shown to increase the sensitivity of cells to chemotherapy [42–44, 58–60]. This effect is driven by various mechanisms, such as the depletion of enzymes that metabolize chemotherapeutics into inactive metabolites [42], or the downregulation of genes associated with chemotherapy resistance [59]. While we observed synergy between tretinoin and CY in vivo, this was not observed in vitro. The sensitivity of AB1HA to CY, or the CY metabolites acrolein and mafosfamide was unaffected by the addition of tretinoin.

Recent research has focussed on identifying biomarkers for response to chemotherapy, to better predict whether patients will respond to treatment or whether they should be given an alternative therapy. An inflammatory, IFN and T cell driven TME is associated with response to not only CY [8], but a range of other chemotherapies [9, 10, 61]. While this signature could be used as a predictive biomarker, an alternative approach is to therapeutically induce this sensitive TME to make subsequent chemotherapy more effective. Tretinoin induces an inflammatory, IFNy-rich TME [12] and was predicted to induce this same CY-sensitive TME. We found that the gene signature associated with tretinoin treatment could separate complete responders from progressors using unsupervised clustering and gene set enrichment, with the pre-treatment TME of complete responders enriched for the tretinoin gene signature. This observation can be exploited by pre-treating tumors with tretinoin to induce the expression of these CY response associated genes, and thereby sensitize the tumor, prior to chemotherapy.

Similar to our findings, it has been reported that tretinoin induced CD8^+^ T cell infiltration in B16 melanoma and that the efficacy of topical tretinoin treatment in controlling tumor growth was partly CD8^+^ T cell dependent [17]. Ablation of RAR signalling in CD8^+^ T cells decreased the accumulation of antigen specific T cells in B16 melanoma [31]. Treatment with tretinoin induces a robust anti-tumor CD8^+^ T cell response, increasing proliferation, expression of effector molecules IFNγ, granzyme B and perforin, as well as activation and cell migration markers [17, 18]. Similar results were observed clinically, with tretinoin increasing antigen specific T cell responses to tumor antigens or vaccination [25, 26].

While we observed robust synergy between tretinoin and CY in AB1-HA, this was not replicated in other cell lines, indicating the phenomenon may be AB1-HAspecific. To investigate what might contribute to this difference in response, we characterised the expression of RARs and RXRs in tumours derived from three cell lines– the sensitive AB1-HA and the resistant AE17 and WEHI164– using RNA sequencing. AB1-HA tumors had increased expression of RARβ and RXRγ. RARβ has been linked to sensitivty to tretinoin. Human colon cancer cell lines with increased expression of RARβ were more sensitive to tretinoin induced apoptosis [16]. Transfection to induce RARβ expression in resistant cell lines increased tretinoin induced cytotoxicty and the expression of pro-apoptotic proteins. While we cannot specifically determine which cells express each of the RARs and RXRs in our model from bulk RNAseq data, in this model the increased presence of RARβ in AB1-HA potentially increases the effects of tretinoin and subsequent downstream effects on CY sensitivity.

Another factor that could contribute towards the differning sensitivity of each tumor model to treatment is the characteristics of the TME. As more inflammatory and immune infiltrated tumors are generally morre sensitive to chemotherapy [8, 48], we investigated whethere there were distinct differences between the CY + Tretinoin responsive AB1-HA and the unresponsive AE17, CT26 and WEHI using previously generated sequencing data collected from each model [8, 36, 37]. Interestingly, treatment sensitive AB1-HA tumors had lower expression of inflammatory genes compared to all other cell lines, suggesting that a pre-treatment inflammatory TME is not the driving force behind this senstivity.

Previous studies have examined the immune composition of these models; AB1-HA is predominantly comprised of myeloid cells with minimal DCs and skewed towards CD4^+^ then CD8^+^ T cells [8]; AE17 is has high levels of macrophages, monocytes and DCs and more CD8^+^ then CD4^+^ T cells [36]; WEHI has similarly a high proportion of macrophages and monocytes but few DCs and Tregs; and CT26 has the highest proportion of DCs and CD8s [37]. It is possible that targeted effects of tretinoin and/or CY on one of these immune cell subsets is responsible for the observed beneficial interaction in the AB1-HA model specifically.

It is important to acknowledge that the data used to identify drugs that are upstream regulators of a CY sensitive TME was generated from the AB1-HA murine mesothelioma tumor model. As the combination of tretinoin and CY was only effective in this model, it may indicate there are limitations in using a single tumor model to predict novel effective drug combinations. As each tumor model differs in immune and stromal composition, vasculature and genetics/transcriptomics (as demonstrated by the difference in RAR and RXR expression in tumors) it may be necessary to generate data from multiple tumor models and identify the common genes and pathways that drive CY sensitivity. Indeed, in other studies we used multiple mouse models to generate response-associated signatures, which we could validate in multiple other tumour models [62, 63]. This suggests that future studies should exclude model-specific biology by deriving the primary input data from multiple models, in order to increase the likelihood that this data translates to other models and, ultimately, to patients.

## Conclusions

Given the excellent safety profile of tretinoin, it is an attractive drug to repurpose for sensitizing cancers to cyclophosphamide chemotherapy, but given the the high variability in efficacy between tumor models without a clear underlying mechanistic explanation, translation into the clinic appears premature at this stage.

### Electronic supplementary material

Below is the link to the electronic supplementary material.

Supplementary Material 1

## Data Availability

The datasets analysed during the current study are available from the GEO repository under accession numbers GSE186195, GSE182674, GSE180618 and GSE229021. A geneset was curated from data generated by Kang et al. [40].

## Abbreviations

CY: Cyclophosphamide
TME: tumor microenvironment
ICT: immune checkpoint therapy
RAR: retinoic acid receptor
Tregs: T regulatory cells
GSEA: Gene set enrichment
LLC: lewis lung carcinoma
RXR: retinoid X receptors
