# Supplementary material for: Tretinoin improves the anti-cancer response to cyclophosphamide, in a model-selective manner
Source: BMC Cancer. 2024 Feb 13;24:203. doi: 10.1186/s12885-024-11915-5 (PMC10865642; doi:10.1186/s12885-024-11915-5)
Supplement: Supplementary file 1 — Supplementary Material 1 [file 12885_2024_11915_MOESM1_ESM.docx]

**SUPPLEMENTARY DATA**

**Supplementary table 1. Survival of mice as determined by the Log-Rank test.**

| Cell Line | CY v PBS | CY v TRETINOIN+CY |
| --- | --- | --- |
| CT26 | 0.0024 | 0.7753 |
| WEHI164 | 0.0026 | 0.1283 |
| 4T1 | 0.0026 | 0.4527 |
| AE17 | 0.0016 | 0.9104 |
| LLC | 0.0175 | 0.9720 |


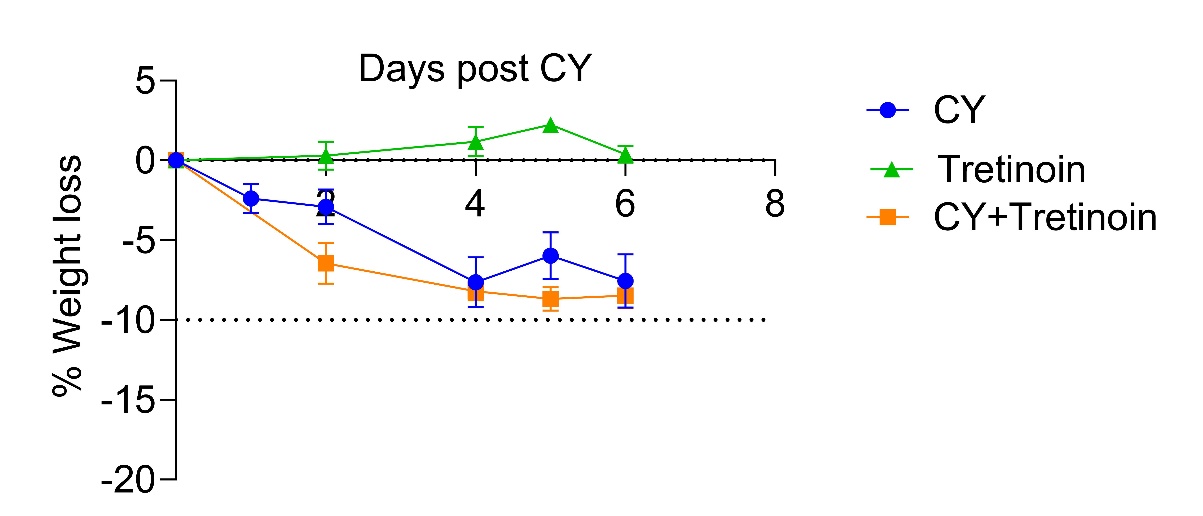


**Supplementary figure 1. There is no additional toxicity with the addition of tretinoin to CY**. Weight loss (%) of mice treated with CY, Tretinoin or the combination.
